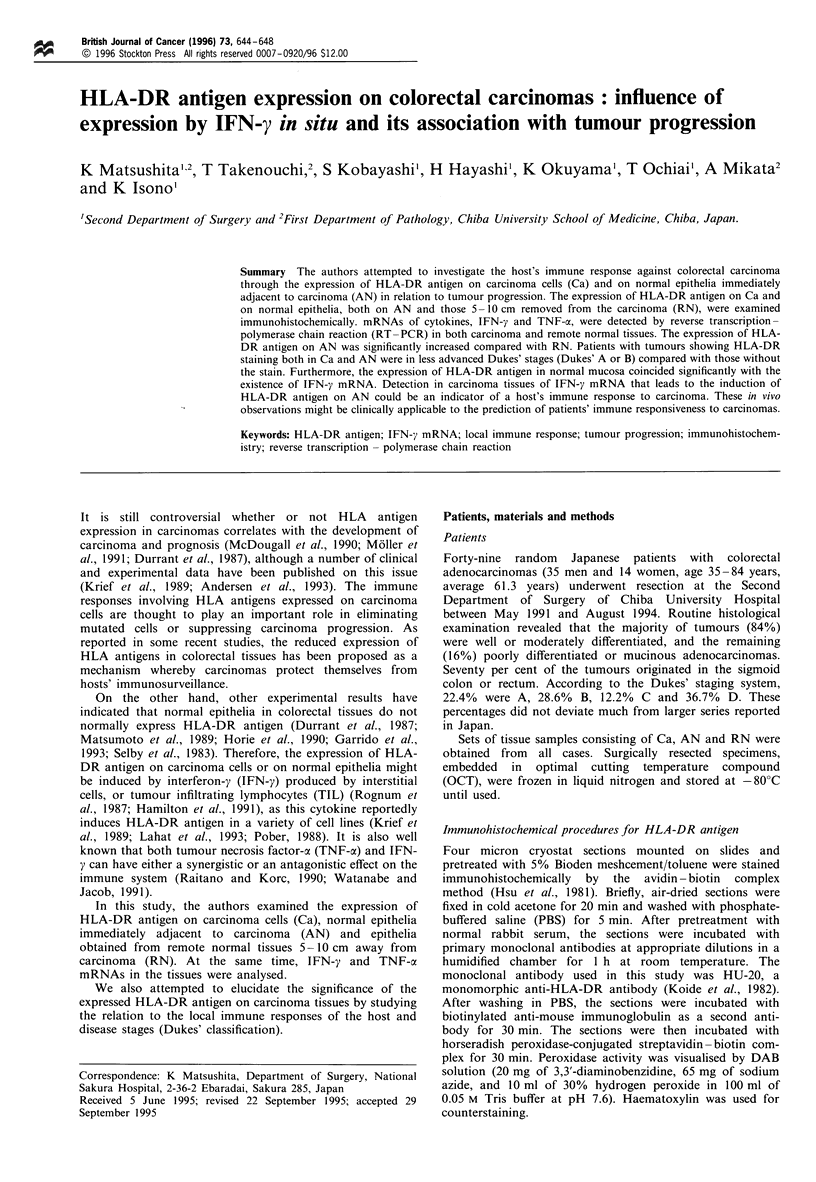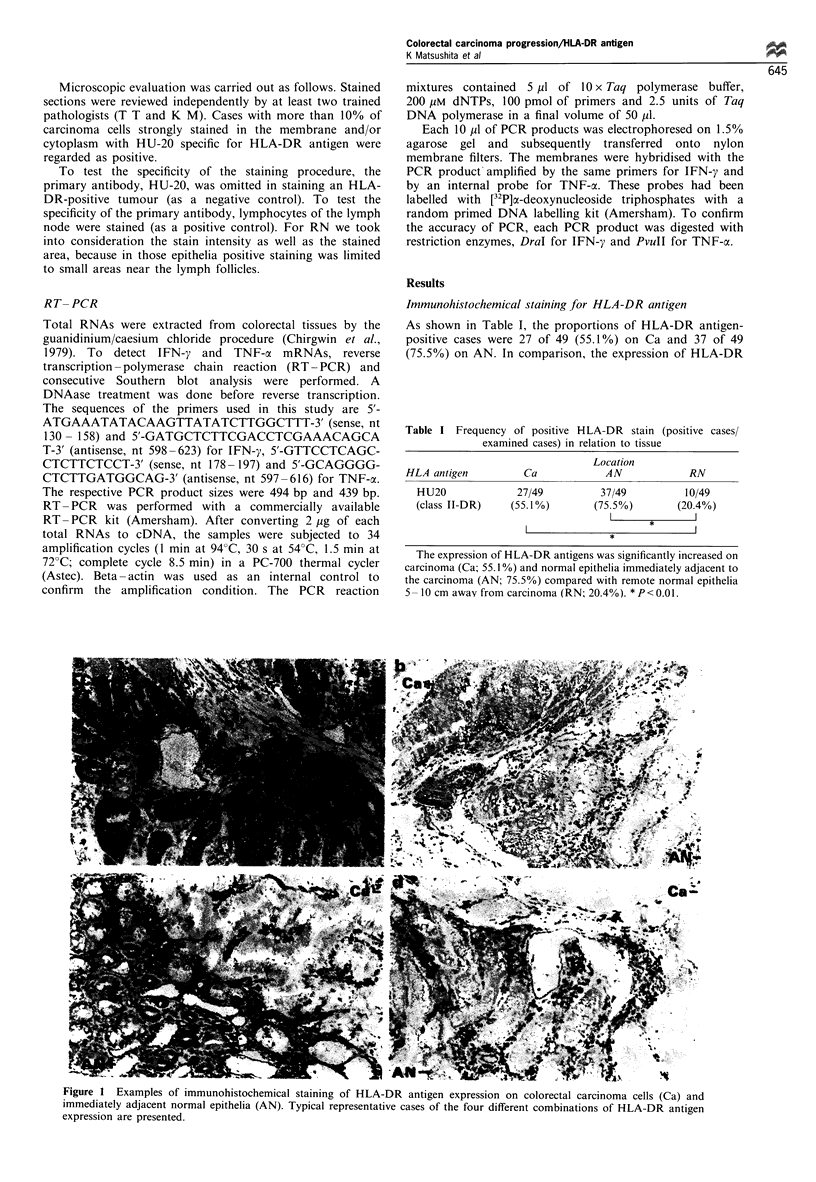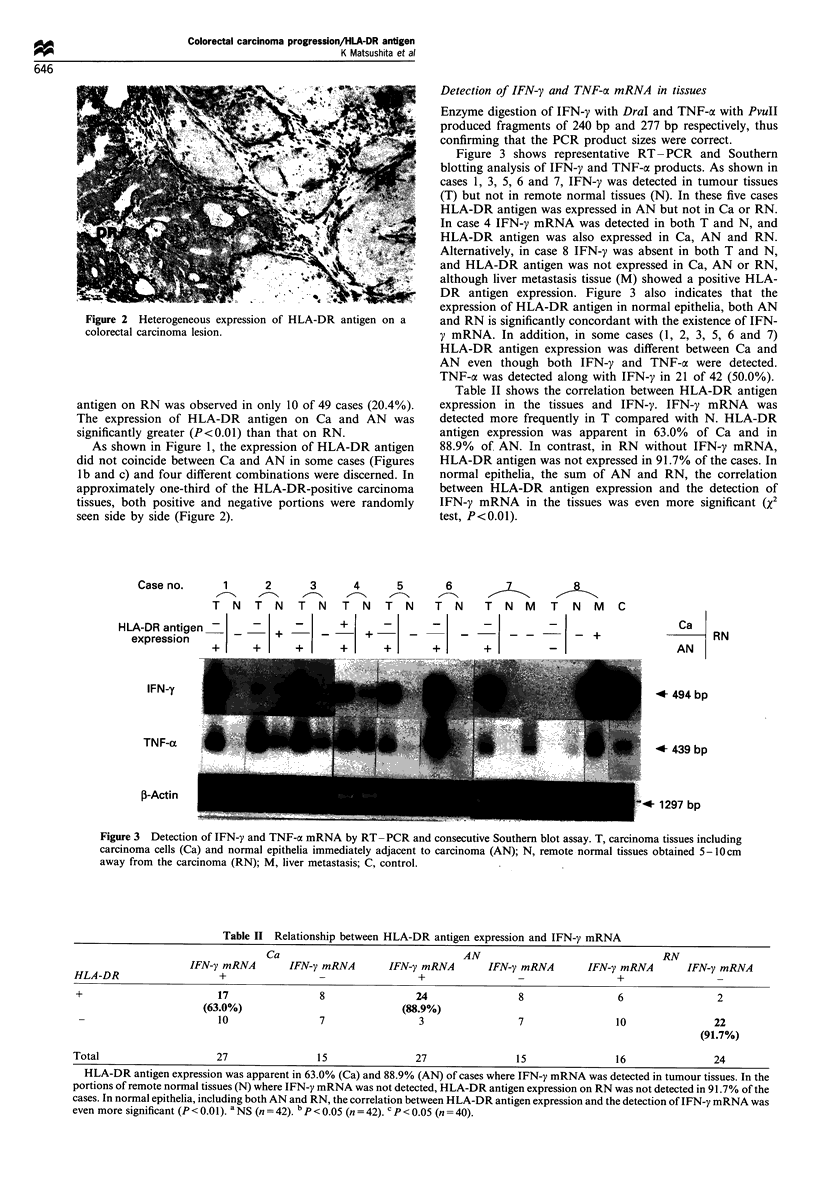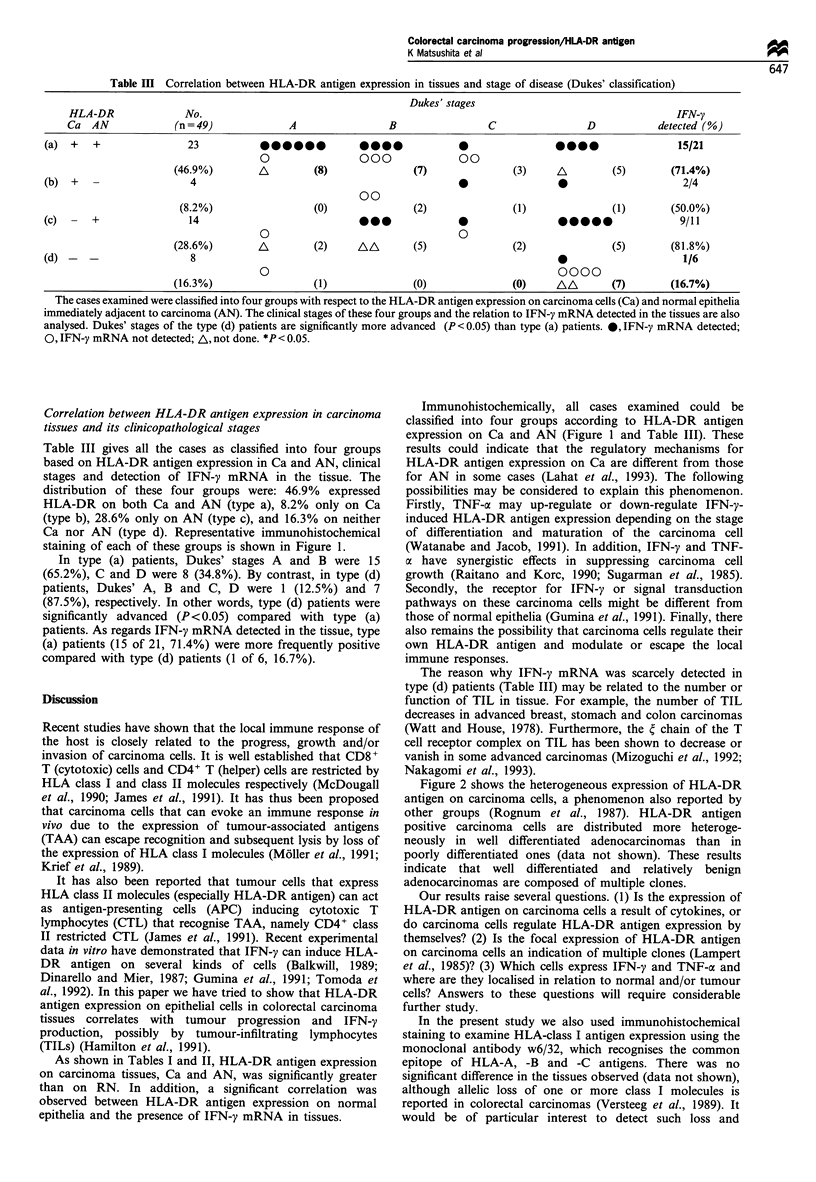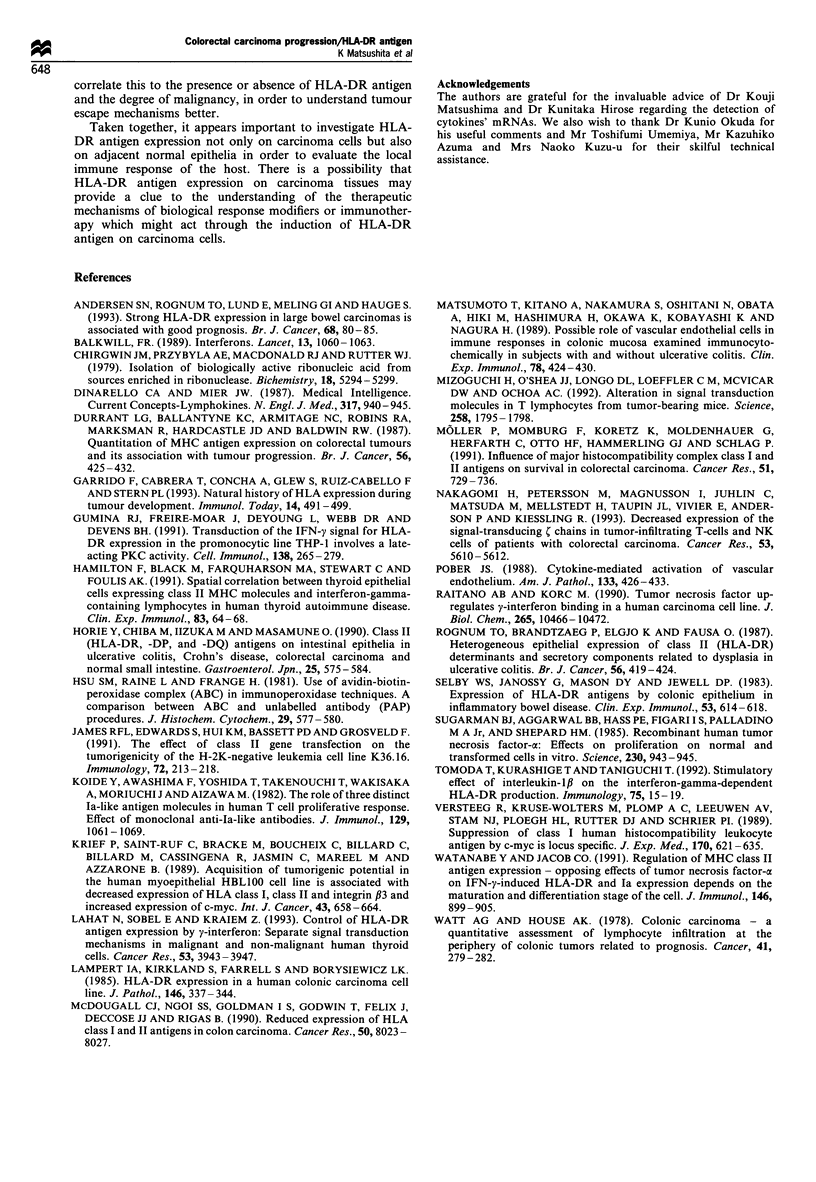# HLA-DR antigen expression in colorectal carcinomas: influence of expression by IFN-gamma in situ and its association with tumour progression.

**DOI:** 10.1038/bjc.1996.112

**Published:** 1996-03

**Authors:** K. Matsushita, T. Takenouchi, S. Kobayashi, H. Hayashi, K. Okuyama, T. Ochiai, A. Mikata, K. Isono

**Affiliations:** Second Department of Surgery, Chiba University School of Medicine, Japan.

## Abstract

**Images:**


British Journal of Cancer (1996) 73, 644-648
© 1996 Stockton Press All rights reserved 0007-0920/96 $12.00

### HLA-DR antigen expression on colorectal carcinomas : influence of expression by IFN-γ *in situ* and its association with tumour progression

K Matsushita[1,2], T Takenouchi,[2], S Kobayashi[1], H Hayashi[1], K Okuyama[1], T Ochiai[1], A Mikata[2] and K Isono[1]

[1]*Second Department of Surgery and* [2]*First Department of Pathology, Chiba University School of Medicine, Chiba, Japan.*

**Summary** The authors attempted to investigate the host's immune response against colorectal carcinoma through the expression of HLA-DR antigen on carcinoma cells (Ca) and on normal epithelia immediately adjacent to carcinoma (AN) in relation to tumour progression. The expression of HLA-DR antigen on Ca and on normal epithelia, both on AN and those 5–10 cm removed from the carcinoma (RN), were examined immunohistochemically. mRNAs of cytokines, IFN-γ and TNF-α, were detected by reverse transcription–polymerase chain reaction (RT–PCR) in both carcinoma and remote normal tissues. The expression of HLA-DR antigen on AN was significantly increased compared with RN. Patients with tumours showing HLA-DR staining both in Ca and AN were in less advanced Dukes' stages (Dukes' A or B) compared with those without the stain. Furthermore, the expression of HLA-DR antigen in normal mucosa coincided significantly with the existence of IFN-γ mRNA. Detection in carcinoma tissues of IFN-γ mRNA that leads to the induction of HLA-DR antigen on AN could be an indicator of a host's immune response to carcinoma. These *in vivo* observations might be clinically applicable to the prediction of patients' immune responsiveness to carcinomas.

**Keywords:** HLA-DR antigen; IFN-γ mRNA; local immune response; tumour progression; immunohistochemistry; reverse transcription – polymerase chain reaction

It is still controversial whether or not HLA antigen expression in carcinomas correlates with the development of carcinoma and prognosis (McDougall *et al.*, 1990; Möller *et al.*, 1991; Durrant *et al.*, 1987), although a number of clinical and experimental data have been published on this issue (Krief *et al.*, 1989; Andersen *et al.*, 1993). The immune responses involving HLA antigens expressed on carcinoma cells are thought to play an important role in eliminating mutated cells or suppressing carcinoma progression. As reported in some recent studies, the reduced expression of HLA antigens in colorectal tissues has been proposed as a mechanism whereby carcinomas protect themselves from hosts' immunosurveillance.

On the other hand, other experimental results have indicated that normal epithelia in colorectal tissues do not normally express HLA-DR antigen (Durrant *et al.*, 1987; Matsumoto *et al.*, 1989; Horie *et al.*, 1990; Garrido *et al.*, 1993; Selby *et al.*, 1983). Therefore, the expression of HLA-DR antigen on carcinoma cells or on normal epithelia might be induced by interferon-γ (IFN-γ) produced by interstitial cells, or tumour infiltrating lymphocytes (TIL) (Rognum *et al.*, 1987; Hamilton *et al.*, 1991), as this cytokine reportedly induces HLA-DR antigen in a variety of cell lines (Krief *et al.*, 1989; Lahat *et al.*, 1993; Pober, 1988). It is also well known that both tumour necrosis factor-α (TNF-α) and IFN-γ can have either a synergistic or an antagonistic effect on the immune system (Raitano and Korc, 1990; Watanabe and Jacob, 1991).

In this study, the authors examined the expression of HLA-DR antigen on carcinoma cells (Ca), normal epithelia immediately adjacent to carcinoma (AN) and epithelia obtained from remote normal tissues 5–10 cm away from carcinoma (RN). At the same time, IFN-γ and TNF-α mRNAs in the tissues were analysed.

We also attempted to elucidate the significance of the expressed HLA-DR antigen on carcinoma tissues by studying the relation to the local immune responses of the host and disease stages (Dukes' classification).

Correspondence: K Matsushita, Department of Surgery, National Sakura Hospital, 2-36-2 Ebaradai, Sakura 285, Japan
Received 5 June 1995; revised 22 September 1995; accepted 29 September 1995

### Patients, materials and methods

### *Patients*

Forty-nine random Japanese patients with colorectal adenocarcinomas (35 men and 14 women, age 35–84 years, average 61.3 years) underwent resection at the Second Department of Surgery of Chiba University Hospital between May 1991 and August 1994. Routine histological examination revealed that the majority of tumours (84%) were well or moderately differentiated, and the remaining (16%) poorly differentiated or mucinous adenocarcinomas. Seventy per cent of the tumours originated in the sigmoid colon or rectum. According to the Dukes' staging system, 22.4% were A, 28.6% B, 12.2% C and 36.7% D. These percentages did not deviate much from larger series reported in Japan.

Sets of tissue samples consisting of Ca, AN and RN were obtained from all cases. Surgically resected specimens, embedded in optimal cutting temperature compound (OCT), were frozen in liquid nitrogen and stored at −80°C until used.

### *Immunohistochemical procedures for HLA-DR antigen*

Four micron cryostat sections mounted on slides and pretreated with 5% Bioden meshcement/toluene were stained immunohistochemically by the avidin–biotin complex method (Hsu *et al.*, 1981). Briefly, air-dried sections were fixed in cold acetone for 20 min and washed with phosphate-buffered saline (PBS) for 5 min. After pretreatment with normal rabbit serum, the sections were incubated with primary monoclonal antibodies at appropriate dilutions in a humidified chamber for 1 h at room temperature. The monoclonal antibody used in this study was HU-20, a monomorphic anti-HLA-DR antibody (Koide *et al.*, 1982). After washing in PBS, the sections were incubated with biotinylated anti-mouse immunoglobulin as a second antibody for 30 min. The sections were then incubated with horseradish peroxidase-conjugated streptavidin–biotin complex for 30 min. Peroxidase activity was visualised by DAB solution (20 mg of 3,3′-diaminobenzidine, 65 mg of sodium azide, and 10 ml of 30% hydrogen peroxide in 100 ml of 0.05 M Tris buffer at pH 7.6). Haematoxylin was used for counterstaining.

Microscopic evaluation was carried out as follows. Stained sections were reviewed independently by at least two trained pathologists (T T and K M). Cases with more than 10% of carcinoma cells strongly stained in the membrane and/or cytoplasm with HU-20 specific for HLA-DR antigen were regarded as positive.

To test the specificity of the staining procedure, the primary antibody, HU-20, was omitted in staining an HLA-DR-positive tumour (as a negative control). To test the specificity of the primary antibody, lymphocytes of the lymph node were stained (as a positive control). For RN we took into consideration the stain intensity as well as the stained area, because in those epithelia positive staining was limited to small areas near the lymph follicles.

### *RT–PCR*

Total RNAs were extracted from colorectal tissues by the guanidinium/caesium chloride procedure (Chirgwin *et al.*, 1979). To detect IFN-$\gamma$ and TNF-$\alpha$ mRNAs, reverse transcription–polymerase chain reaction (RT–PCR) and consecutive Southern blot analysis were performed. A DNAase treatment was done before reverse transcription. The sequences of the primers used in this study are 5′-ATGAAATATACAAGTTATATCTTGGCTTT-3′ (sense, nt 130 – 158) and 5′-GATGCTCTTCGACCTCGAAACAGCAT-3′ (antisense, nt 598–623) for IFN-$\gamma$, 5′-GTTCCTCAGCCTCTTCTCCT-3′ (sense, nt 178–197) and 5′-GCAGGGGCTCTTGATGGCAG-3′ (antisense, nt 597–616) for TNF-$\alpha$. The respective PCR product sizes were 494 bp and 439 bp. RT–PCR was performed with a commercially available RT–PCR kit (Amersham). After converting 2 $\mu$g of each total RNAs to cDNA, the samples were subjected to 34 amplification cycles (1 min at 94°C, 30 s at 54°C, 1.5 min at 72°C; complete cycle 8.5 min) in a PC-700 thermal cycler (Astec). Beta–actin was used as an internal control to confirm the amplification condition. The PCR reaction mixtures contained 5 $\mu$l of 10 × *Taq* polymerase buffer, 200 $\mu$M dNTPs, 100 pmol of primers and 2.5 units of *Taq* DNA polymerase in a final volume of 50 $\mu$l.

Each 10 $\mu$l of PCR products was electrophoresed on 1.5% agarose gel and subsequently transferred onto nylon membrane filters. The membranes were hybridised with the PCR product amplified by the same primers for IFN-$\gamma$ and by an internal probe for TNF-$\alpha$. These probes had been labelled with [$^{32}$P]$\alpha$-deoxynucleoside triphosphates with a random primed DNA labelling kit (Amersham). To confirm the accuracy of PCR, each PCR product was digested with restriction enzymes, *Dra*I for IFN-$\gamma$ and *Pvu*II for TNF-$\alpha$.

### Results

### *Immunohistochemical staining for HLA-DR antigen*

As shown in Table I, the proportions of HLA-DR antigen-positive cases were 27 of 49 (55.1%) on Ca and 37 of 49 (75.5%) on AN. In comparison, the expression of HLA-DR

**Table I** Frequency of positive HLA-DR stain (positive cases/examined cases) in relation to tissue

| | Location | | |
|---|---|---|---|
| *HLA antigen* | *Ca* | *AN* | *RN* |
| HU20 (class II-DR) | 27/49 (55.1%) | 37/49 (75.5%) | 10/49 (20.4%) |

(AN vs RN: *; Ca vs RN: *)

The expression of HLA-DR antigens was significantly increased on carcinoma (Ca; 55.1%) and normal epithelia immediately adjacent to the carcinoma (AN; 75.5%) compared with remote normal epithelia 5–10 cm away from carcinoma (RN; 20.4%). * $P < 0.01$.

**Figure 1** Examples of immunohistochemical staining of HLA-DR antigen expression on colorectal carcinoma cells (Ca) and immediately adjacent normal epithelia (AN). Typical representative cases of the four different combinations of HLA-DR antigen expression are presented.

**Figure 2** Heterogeneous expression of HLA-DR antigen on a colorectal carcinoma lesion.

antigen on RN was observed in only 10 of 49 cases (20.4%). The expression of HLA-DR antigen on Ca and AN was significantly greater ($P<0.01$) than that on RN.

As shown in Figure 1, the expression of HLA-DR antigen did not coincide between Ca and AN in some cases (Figures 1b and c) and four different combinations were discerned. In approximately one-third of the HLA-DR-positive carcinoma tissues, both positive and negative portions were randomly seen side by side (Figure 2).

*Detection of IFN-γ and TNF-α mRNA in tissues*

Enzyme digestion of IFN-γ with *Dra*I and TNF-α with *Pvu*II produced fragments of 240 bp and 277 bp respectively, thus confirming that the PCR product sizes were correct.

Figure 3 shows representative RT–PCR and Southern blotting analysis of IFN-γ and TNF-α products. As shown in cases 1, 3, 5, 6 and 7, IFN-γ was detected in tumour tissues (T) but not in remote normal tissues (N). In these five cases HLA-DR antigen was expressed in AN but not in Ca or RN. In case 4 IFN-γ mRNA was detected in both T and N, and HLA-DR antigen was also expressed in Ca, AN and RN. Alternatively, in case 8 IFN-γ was absent in both T and N, and HLA-DR antigen was not expressed in Ca, AN or RN, although liver metastasis tissue (M) showed a positive HLA-DR antigen expression. Figure 3 also indicates that the expression of HLA-DR antigen in normal epithelia, both AN and RN is significantly concordant with the existence of IFN-γ mRNA. In addition, in some cases (1, 2, 3, 5, 6 and 7) HLA-DR antigen expression was different between Ca and AN even though both IFN-γ and TNF-α were detected. TNF-α was detected along with IFN-γ in 21 of 42 (50.0%).

Table II shows the correlation between HLA-DR antigen expression in the tissues and IFN-γ. IFN-γ mRNA was detected more frequently in T compared with N. HLA-DR antigen expression was apparent in 63.0% of Ca and in 88.9% of AN. In contrast, in RN without IFN-γ mRNA, HLA-DR antigen was not expressed in 91.7% of the cases. In normal epithelia, the sum of AN and RN, the correlation between HLA-DR antigen expression and the detection of IFN-γ mRNA in the tissues was even more significant ($\chi^2$ test, $P<0.01$).

**Figure 3** Detection of IFN-γ and TNF-α mRNA by RT–PCR and consecutive Southern blot assay. T, carcinoma tissues including carcinoma cells (Ca) and normal epithelia immediately adjacent to carcinoma (AN); N, remote normal tissues obtained 5–10 cm away from the carcinoma (RN); M, liver metastasis; C, control.

**Table II** Relationship between HLA-DR antigen expression and IFN-γ mRNA

| HLA-DR | *Ca* IFN-γ mRNA + | *Ca* IFN-γ mRNA − | *AN* IFN-γ mRNA + | *AN* IFN-γ mRNA − | *RN* IFN-γ mRNA + | *RN* IFN-γ mRNA − |
|---|---|---|---|---|---|---|
| + | **17 (63.0%)** | 8 | **24 (88.9%)** | 8 | 6 | 2 |
| − | 10 | 7 | 3 | 7 | 10 | **22 (91.7%)** |
| Total | 27 | 15 | 27 | 15 | 16 | 24 |

HLA-DR antigen expression was apparent in 63.0% (Ca) and 88.9% (AN) of cases where IFN-γ mRNA was detected in tumour tissues. In the portions of remote normal tissues (N) where IFN-γ mRNA was not detected, HLA-DR antigen expression on RN was not detected in 91.7% of the cases. In normal epithelia, including both AN and RN, the correlation between HLA-DR antigen expression and the detection of IFN-γ mRNA was even more significant ($P<0.01$). [a]NS ($n=42$). [b]$P<0.05$ ($n=42$). [c]$P<0.05$ ($n=40$).

**Table III** Correlation between HLA-DR antigen expression in tissues and stage of disease (Dukes' classification)

| | *HLA-DR* Ca | *HLA-DR* AN | *No.* (n=49) | *Dukes' stages* A | B | C | D | *IFN-γ detected (%)* |
|---|---|---|---|---|---|---|---|---|
| (a) | + | + | 23<br>(46.9%) | ●●●●●●<br>○<br>△ **(8)** | ●●●●<br>○○○<br>**(7)** | ●<br>○○<br>(3) | ●●●●<br>△ (5) | **15/21**<br>**(71.4%)** |
| (b) | + | − | 4<br>(8.2%) | (0) | ○○<br>(2) | ●<br>(1) | ●<br>(1) | 2/4<br>(50.0%) |
| (c) | − | + | 14<br>(28.6%) | ○<br>△ (2) | ●●●<br>△△ (5) | ●<br>○<br>(2) | ●●●●●<br>(5) | 9/11<br>(81.8%) |
| (d) | — | — | 8<br>(16.3%) | ○<br>(1) | (0) | **(0)** | ●<br>○○○○<br>△△ **(7)** | **1/6**<br>**(16.7%)** |

The cases examined were classified into four groups with respect to the HLA-DR antigen expression on carcinoma cells (Ca) and normal epithelia immediately adjacent to carcinoma (AN). The clinical stages of these four groups and the relation to IFN-γ mRNA detected in the tissues are also analysed. Dukes' stages of the type (d) patients are significantly more advanced ($P<0.05$) than type (a) patients. ●, IFN-γ mRNA detected; ○, IFN-γ mRNA not detected; △, not done. *$P<0.05$.

*Correlation between HLA-DR antigen expression in carcinoma tissues and its clinicopathological stages*

Table III gives all the cases as classified into four groups based on HLA-DR antigen expression in Ca and AN, clinical stages and detection of IFN-γ mRNA in the tissue. The distribution of these four groups were: 46.9% expressed HLA-DR on both Ca and AN (type a), 8.2% only on Ca (type b), 28.6% only on AN (type c), and 16.3% on neither Ca nor AN (type d). Representative immunohistochemical staining of each of these groups is shown in Figure 1.

In type (a) patients, Dukes' stages A and B were 15 (65.2%), C and D were 8 (34.8%). By contrast, in type (d) patients, Dukes' A, B and C, D were 1 (12.5%) and 7 (87.5%), respectively. In other words, type (d) patients were significantly advanced ($P<0.05$) compared with type (a) patients. As regards IFN-γ mRNA detected in the tissue, type (a) patients (15 of 21, 71.4%) were more frequently positive compared with type (d) patients (1 of 6, 16.7%).

### Discussion

Recent studies have shown that the local immune response of the host is closely related to the progress, growth and/or invasion of carcinoma cells. It is well established that $CD8^+$ T (cytotoxic) cells and $CD4^+$ T (helper) cells are restricted by HLA class I and class II molecules respectively (McDougall *et al.*, 1990; James *et al.*, 1991). It has thus been proposed that carcinoma cells that can evoke an immune response *in vivo* due to the expression of tumour-associated antigens (TAA) can escape recognition and subsequent lysis by loss of the expression of HLA class I molecules (Möller *et al.*, 1991; Krief *et al.*, 1989).

It has also been reported that tumour cells that express HLA class II molecules (especially HLA-DR antigen) can act as antigen-presenting cells (APC) inducing cytotoxic T lymphocytes (CTL) that recognise TAA, namely $CD4^+$ class II restricted CTL (James *et al.*, 1991). Recent experimental data *in vitro* have demonstrated that IFN-γ can induce HLA-DR antigen on several kinds of cells (Balkwill, 1989; Dinarello and Mier, 1987; Gumina *et al.*, 1991; Tomoda *et al.*, 1992). In this paper we have tried to show that HLA-DR antigen expression on epithelial cells in colorectal carcinoma tissues correlates with tumour progression and IFN-γ production, possibly by tumour-infiltrating lymphocytes (TILs) (Hamilton *et al.*, 1991).

As shown in Tables I and II, HLA-DR antigen expression on carcinoma tissues, Ca and AN, was significantly greater than on RN. In addition, a significant correlation was observed between HLA-DR antigen expression on normal epithelia and the presence of IFN-γ mRNA in tissues.

Immunohistochemically, all cases examined could be classified into four groups according to HLA-DR antigen expression on Ca and AN (Figure 1 and Table III). These results could indicate that the regulatory mechanisms for HLA-DR antigen expression on Ca are different from those for AN in some cases (Lahat *et al.*, 1993). The following possibilities may be considered to explain this phenomenon. Firstly, TNF-α may up-regulate or down-regulate IFN-γ-induced HLA-DR antigen expression depending on the stage of differentiation and maturation of the carcinoma cell (Watanabe and Jacob, 1991). In addition, IFN-γ and TNF-α have synergistic effects in suppressing carcinoma cell growth (Raitano and Korc, 1990; Sugarman *et al.*, 1985). Secondly, the receptor for IFN-γ or signal transduction pathways on these carcinoma cells might be different from those of normal epithelia (Gumina *et al.*, 1991). Finally, there also remains the possibility that carcinoma cells regulate their own HLA-DR antigen and modulate or escape the local immune responses.

The reason why IFN-γ mRNA was scarcely detected in type (d) patients (Table III) may be related to the number or function of TIL in tissue. For example, the number of TIL decreases in advanced breast, stomach and colon carcinomas (Watt and House, 1978). Furthermore, the ξ chain of the T cell receptor complex on TIL has been shown to decrease or vanish in some advanced carcinomas (Mizoguchi *et al.*, 1992; Nakagomi *et al.*, 1993).

Figure 2 shows the heterogeneous expression of HLA-DR antigen on carcinoma cells, a phenomenon also reported by other groups (Rognum *et al.*, 1987). HLA-DR antigen positive carcinoma cells are distributed more heterogeneously in well differentiated adenocarcinomas than in poorly differentiated ones (data not shown). These results indicate that well differentiated and relatively benign adenocarcinomas are composed of multiple clones.

Our results raise several questions. (1) Is the expression of HLA-DR antigen on carcinoma cells a result of cytokines, or do carcinoma cells regulate HLA-DR antigen expression by themselves? (2) Is the focal expression of HLA-DR antigen on carcinoma cells an indication of multiple clones (Lampert *et al.*, 1985)? (3) Which cells express IFN-γ and TNF-α and where are they localised in relation to normal and/or tumour cells? Answers to these questions will require considerable further study.

In the present study we also used immunohistochemical staining to examine HLA-class I antigen expression using the monoclonal antibody w6/32, which recognises the common epitope of HLA-A, -B and -C antigens. There was no significant difference in the tissues observed (data not shown), although allelic loss of one or more class I molecules is reported in colorectal carcinomas (Versteeg *et al.*, 1989). It would be of particular interest to detect such loss and correlate this to the presence or absence of HLA-DR antigen and the degree of malignancy, in order to understand tumour escape mechanisms better.

Taken together, it appears important to investigate HLA-DR antigen expression not only on carcinoma cells but also on adjacent normal epithelia in order to evaluate the local immune response of the host. There is a possibility that HLA-DR antigen expression on carcinoma tissues may provide a clue to the understanding of the therapeutic mechanisms of biological response modifiers or immunotherapy which might act through the induction of HLA-DR antigen on carcinoma cells.

**Acknowledgements**
The authors are grateful for the invaluable advice of Dr Kouji Matsushima and Dr Kunitaka Hirose regarding the detection of cytokines' mRNAs. We also wish to thank Dr Kunio Okuda for his useful comments and Mr Toshifumi Umemiya, Mr Kazuhiko Azuma and Mrs Naoko Kuzu-u for their skilful technical assistance.

### References

ANDERSEN SN, ROGNUM TO, LUND E, MELING GI AND HAUGE S. (1993). Strong HLA-DR expression in large bowel carcinomas is associated with good prognosis. *Br. J. Cancer*, **68**, 80–85.

BALKWILL, FR. (1989). Interferons. *Lancet*, **13**, 1060–1063.

CHIRGWIN JM, PRZYBYLA AE, MACDONALD RJ AND RUTTER WJ. (1979). Isolation of biologically active ribonucleic acid from sources enriched in ribonuclease. *Bichemistry*, **18**, 5294–5299.

DINARELLO CA AND MIER JW. (1987). Medical Intelligence. Current Concepts-Lymphokines. *N. Engl. J. Med.*, **317**, 940–945.

DURRANT LG, BALLANTYNE KC, ARMITAGE NC, ROBINS RA, MARKSMAN R, HARDCASTLE JD AND BALDWIN RW. (1987). Quantitation of MHC antigen expression on colorectal tumours and its association with tumour progression. *Br. J. Cancer*, **56**, 425–432.

GARRIDO F, CABRERA T, CONCHA A, GLEW S, RUIZ-CABELLO F AND STERN PL (1993). Natural history of HLA expression during tumour development. *Immunol. Today*, **14**, 491–499.

GUMINA RJ, FREIRE-MOAR J, DEYOUNG L, WEBB DR AND DEVENS BH. (1991). Transduction of the IFN-$\gamma$ signal for HLA-DR expression in the promonocytic line THP-1 involves a late-acting PKC activity. *Cell. Immunol.*, **138**, 265–279.

HAMILTON F, BLACK M, FARQUHARSON MA, STEWART C AND FOULIS AK. (1991). Spatial correlation between thyroid epithelial cells expressing class II MHC molecules and interferon-gamma-containing lymphocytes in human thyroid autoimmune disease. *Clin. Exp. Immunol.*, **83**, 64–68.

HORIE Y, CHIBA M, IIZUKA M AND MASAMUNE O. (1990). Class II (HLA-DR, -DP, and -DQ) antigens on intestinal epithelia in ulcerative colitis, Crohn's disease, colorectal carcinoma and normal small intestine. *Gastroenterol. Jpn.*, **25**, 575–584.

HSU SM, RAINE L AND FRANGE H. (1981). Use of avidin-biotin-peroxidase complex (ABC) in immunoperoxidase techniques. A comparison between ABC and unlabelled antibody (PAP) procedures. *J. Histochem. Cytochem.*, **29**, 577–580.

JAMES RFL, EDWARDS S, HUI KM, BASSETT PD AND GROSVELD F. (1991). The effect of class II gene transfection on the tumorigenicity of the H-2K-negative leukemia cell line K36.16. *Immunology*, **72**, 213–218.

KOIDE Y, AWASHIMA F, YOSHIDA T, TAKENOUCHI T, WAKISAKA A, MORIUCHI J AND AIZAWA M. (1982). The role of three distinct Ia-like antigen molecules in human T cell proliferative response. Effect of monoclonal anti-Ia-like antibodies. *J. Immunol.*, **129**, 1061–1069.

KRIEF P, SAINT-RUF C, BRACKE M, BOUCHEIX C, BILLARD C, BILLARD M, CASSINGENA R, JASMIN C, MAREEL M AND AZZARONE B. (1989). Acquisition of tumorigenic potential in the human myoepithelial HBL100 cell line is associated with decreased expression of HLA class I, class II and integrin $\beta 3$ and increased expression of c-myc. *Int. J. Cancer*, **43**, 658–664.

LAHAT N, SOBEL E AND KRAIEM Z. (1993). Control of HLA-DR antigen expression by $\gamma$-interferon: Separate signal transduction mechanisms in malignant and non-malignant human thyroid cells. *Cancer Res.*, **53**, 3943–3947.

LAMPERT IA, KIRKLAND S, FARRELL S AND BORYSIEWICZ LK. (1985). HLA-DR expression in a human colonic carcinoma cell line. *J. Pathol.*, **146**, 337–344.

McDOUGALL CJ, NGOI SS, GOLDMAN I S, GODWIN T, FELIX J, DECCOSE JJ AND RIGAS B. (1990). Reduced expression of HLA class I and II antigens in colon carcinoma. *Cancer Res.*, **50**, 8023–8027.

MATSUMOTO T, KITANO A, NAKAMURA S, OSHITANI N, OBATA A, HIKI M, HASHIMURA H, OKAWA K, KOBAYASHI K AND NAGURA H. (1989). Possible role of vascular endothelial cells in immune responses in colonic mucosa examined immunocytochemically in subjects with and without ulcerative colitis. *Clin. Exp. Immunol.*, **78**, 424–430.

MIZOGUCHI H, O'SHEA JJ, LONGO DL, LOEFFLER C M, MCVICAR DW AND OCHOA AC. (1992). Alteration in signal transduction molecules in T lymphocytes from tumor-bearing mice. *Science*, **258**, 1795–1798.

MÕLLER P, MOMBURG F, KORETZ K, MOLDENHAUER G, HERFARTH C, OTTO HF, HAMMERLING GJ AND SCHLAG P. (1991). Influence of major histocompatibility complex class I and II antigens on survival in colorectal carcinoma. *Cancer Res.*, **51**, 729–736.

NAKAGOMI H, PETERSSON M, MAGNUSSON I, JUHLIN C, MATSUDA M, MELLSTEDT H, TAUPIN JL, VIVIER E, ANDERSON P AND KIESSLING R. (1993). Decreased expression of the signal-transducing $\zeta$ chains in tumor-infiltrating T-cells and NK cells of patients with colorectal carcinoma. *Cancer Res.*, **53**, 5610–5612.

POBER JS. (1988). Cytokine-mediated activation of vascular endothelium. *Am. J. Pathol.*, **133**, 426–433.

RAITANO AB AND KORC M. (1990). Tumor necrosis factor up-regulates $\gamma$-interferon binding in a human carcinoma cell line. *J. Biol. Chem.*, **265**, 10466–10472.

ROGNUM TO, BRANDTZAEG P, ELGJO K AND FAUSA O. (1987). Heterogeneous epithelial expression of class II (HLA-DR) determinants and secretory components related to dysplasia in ulcerative colitis. *Br. J. Cancer*, **56**, 419–424.

SELBY WS, JANOSSY G, MASON DY AND JEWELL DP. (1983). Expression of HLA-DR antigens by colonic epithelium in inflammatory bowel disease. *Clin. Exp. Immunol.*, **53**, 614–618.

SUGARMAN BJ, AGGARWAL BB, HASS PE, FIGARI I S, PALLADINO M A Jr, AND SHEPARD HM. (1985). Recombinant human tumor necrosis factor-$\alpha$: Effects on proliferation on normal and transformed cells in vitro. *Science*, **230**, 943–945.

TOMODA T, KURASHIGE T AND TANIGUCHI T. (1992). Stimulatory effect of interleukin-1$\beta$ on the interferon-gamma-dependent HLA-DR production. *Immunology*, **75**, 15–19.

VERSTEEG R, KRUSE-WOLTERS M, PLOMP A C, LEEUWEN AV, STAM NJ, PLOEGH HL, RUTTER DJ AND SCHRIER PI. (1989). Suppression of class I human histocompatibility leukocyte antigen by c-myc is locus specific. *J. Exp. Med.*, **170**, 621–635.

WATANABE Y AND JACOB CO. (1991). Regulation of MHC class II antigen expression – opposing effects of tumor necrosis factor-$\alpha$ on IFN-$\gamma$-induced HLA-DR and Ia expression depends on the maturation and differentiation stage of the cell. *J. Immunol.*, **146**, 899–905.

WATT AG AND HOUSE AK. (1978). Colonic carcinoma – a quantitative assessment of lymphocyte infiltration at the periphery of colonic tumors related to prognosis. *Cancer*, **41**, 279–282.